# A protocol for resuscitation of severe burn patients guided by transpulmonary thermodilution and lactate levels: a 3-year prospective cohort study

**DOI:** 10.1186/cc12855

**Published:** 2013-08-15

**Authors:** Manuel Sánchez, Abelardo García-de-Lorenzo, Eva Herrero, Teresa Lopez, Beatriz Galvan, María José Asensio, Lucia Cachafeiro, Cesar Casado

**Affiliations:** 1 Unidad de Quemados Críticos, Servicio de Medicina Intensiva, Hospital Universitario La Paz, Paseo de la Castellana, n° 261, 28046 Madrid, Spain; 2 Unidad de Ecocardiografia, Servicio de Cardiología, Hospital Universitario La Paz, Paseo de la Castellana, n° 261, 28046 Madrid, Spain; 3 Unidad de Quemados Críticos, Servicio de Cirugía Plástica, Hospital Universitario La Paz, Paseo de la Castellana, n° 261, 28046 Madrid, Spain

## Abstract

**Introduction:**

The use of urinary output and vital signs to guide initial burn resuscitation may lead to suboptimal resuscitation. Invasive hemodynamic monitoring may result in over-resuscitation. This study aimed to evaluate the results of a goal-directed burn resuscitation protocol that used standard measures of mean arterial pressure (MAP) and urine output, plus transpulmonary thermodilution (TPTD) and lactate levels to adjust fluid therapy to achieve a minimum level of preload to allow for sufficient vital organ perfusion.

**Methods:**

We conducted a three-year prospective cohort study of 132 consecutive critically burned patients. These patients underwent resuscitation guided by MAP (>65 mmHg), urinary output (0.5 to 1 ml/kg), TPTD and lactate levels. Fluid therapy was adjusted to achieve a cardiac index (CI) >2.5 L/minute/m^2 ^and an intrathoracic blood volume index (ITBVI) >600 ml/m^2^, and to optimize lactate levels. Statistical analysis was performed using mixed models. We also used Pearson or Spearman methods and the Mann-Whitney U-test.

**Results:**

A total of 98 men and 34 women (mean age, 48 ± 18 years) was studied. The mean total body surface area (TBSA) burned was 35% ± 22%. During the early resuscitation phase, lactate levels were elevated (2.58 ± 2.05 mmol/L) and TPTD showed initial hypovolemia by the CI (2.68 ± 1.06 L/minute/m^2^) and the ITBVI (709 ± 254 mL/m^2^). At 24 to 32 hours, the CI and lactic levels were normalized, although the ITBVI remained below the normal range (744 ± 276 ml/m^2^). The mean fluid rate required to achieve protocol targets in the first 8 hours was 4.05 ml/kg/TBSA burned, which slightly increased in the next 16 hours. Patients with a urine output greater than or less than 0.5 ml/kg/hour did not show differences in heart rate, mean arterial pressure, CI, ITBVI or lactate levels.

**Conclusions:**

Initial hypovolemia may be detected by TPTD monitoring during the early resuscitation phase. This hypovolemia might not be reflected by blood pressure and hourly urine output. An adequate CI and tissue perfusion can be achieved with below-normal levels of preload. Early resuscitation guided by lactate levels and below-normal preload volume targets appears safe and avoids unnecessary fluid input.

## Introduction

Hemodynamic changes during the initial resuscitation phase of critically burned patients are primarily due to intravascular volume loss. Proper replacement improves prognosis [1]. However, in more severe patients, it is difficult to achieve the optimal rate of replenishment to correct hypovolemia without unnecessarily increasing fluid in the interstitial space. This balance is important because elevation of the interstitial fluid may enhance morbidity and mortality by increasing the length of stay and mechanical ventilation time. This causes compartmental syndromes, increasing the depth of the burn, and might cause lung edema [2,3].

Fluid requirement calculations based on Parkland's formula and monitoring with simple data, such as hourly urine output and mean arterial pressure (MAP), are the most widespread approaches for resuscitation; however, it is controversial whether these parameters are sufficient [4-7]. Monitoring of cardiac output (CO) and filling pressures has shown that Parkland's formula is not always able to correct for initial hypovolemia. Therefore, some authors have guided resuscitation according to these parameters, although this approach led them to give potentially excessive volume [5,8-11]. The finding that preload pressures are poor volemic markers has led to increased resuscitation guided by data from the transpulmonary thermodilution (TPTD) method [12,13]. This method measures CO, intrathoracic blood volume (ITBV), extravascular lung water (EVLW) and other dynamic parameters. Previous studies have shown that TPTD monitoring results in more aggressive therapeutic strategies and is associated with a significant increase in fluid administration, but it does not improve preload [14]. In a preliminary study with 24 patients, Arlati *et al*. used a permissive hypovolemia protocol and reduced the volume given as low as possible by titrating the infusion rate to a minimum ITBV value that allowed for at least 2.2 L/minute/m^2 ^of the cardiac index (CI). They found that this protocol was effective in reducing multiple organ dysfunction [15].

Therefore, we designed a permissive hypovolemia protocol with lower preload targets and associated lactate measurements to ensure tissue perfusion. Furthermore, we performed echocardiography and measurements of some useful biomarkers, including lactic acid, which is a global perfusion marker, troponin I, which is a heart injury marker, and N-terminal pro-brain natriuretic peptide (NTproBNP), which is affected by cardiac dysfunction [16-18]. The purpose of this study was to evaluate the results of a resuscitation protocol that used standard measures of mean arterial pressure (MAP) and urine output plus TPTD and lactate levels to adjust fluid therapy to achieve a minimum level of preload to allow for sufficient vital organ perfusion.

Secondary objectives included determining if adequate urine output ensures good resuscitation and determining if normal preload values are required to ensure good resuscitation. Our primary hypothesis was that early resuscitation guided by lactate levels and hemodynamic targets below normal is safe and avoids unnecessary fluid input. Our secondary hypothesis was that initial hypovolemia can be detected by TPTD monitoring during the early resuscitation phase, and this is not reflected by blood pressure and hourly urine output.

## Materials and methods

A prospective study was performed in 362 burn patients admitted to our unit between October 2008 and October 2011. Of these, 132 patients were critically burned (defined as ≥20% of the surface area burned, inhalation syndrome, electrical injury mechanisms, advanced age, or involvement of the head and neck) and did not have exclusion criteria (including pregnancy, age <18 years, interval from burn to monitoring >8 hours, and contraindications for central venous access or femoral arterial line). The protocol was approved by the Clinical Research Ethics Committee of the 'La Paz' University Hospital (Madrid, Spain) and all necessary written and signed consent was obtained from any patients involved in the study, including consent to participate in the study and consent to publish.

Epidemiological data were collected from all patients who were monitored with the PiCCO^® ^system (Pulsion Medical Systems, Munich, Germany) for transpulmonary dilution. Blood samples were obtained to measure biochemical values every 8 hours from admission to 72 hours. Serial echocardiography was also performed.

**Figure 1:**
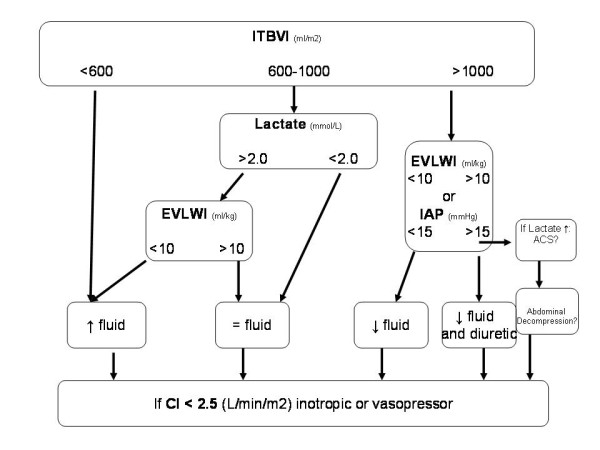
**Resuscitation decision tree**. A diagram of a decision tree for the adjustment of fluid and catecholamine therapy according to a permissive hypovolemia protocol with lower preload targets and lactate measurements to ensure tissue perfusion is shown.

Resuscitation was initiated with Ringer's lactate according to the Parkland formula. Subsequently, the fluid rate was increased when the hourly urine output was <0.5 mL/kg or MAP <65 mm Hg or decreased when the hourly urine output was >1 ml/kg. Re-evaluation according to our protocol (Figure 1) was performed every eight hours and more frequently whenever urine output was below the target for two consecutive hours or MAP was below 55 mm Hg. At 12 to 24 hours, 6% hydroxyethyl starch 130/0.4 (Voluven^® ^Fresenius Kabi, Bad Homburg, Germany) was added, the amount of Ringer's lactate reduced, oral tolerance tested in patients not receiving mechanical ventilation and enteral tolerance tests with water performed every 8 hours in intubated patients. After the second day of admission, each patient received a maintenance fluid volume calculated as 3,000 mL plus the exudates (3,750 mL × BSA (m^2^) × (% burn/100)) and nutritional support. Enteral feeding was used where possible. Patients with high total body surface areas (TBSAs) burned received supplementary nutrition. Patients who did not tolerate enteral nutrition received total parenteral nutrition. Eighty-one patients (61.3%) received total or supplemental parenteral nutrition.

Blood pressure was measured by a femoral arterial catheter, which in addition to a central venous line, was used to perform single TPTD by the PiCCO^® ^technique [19]. This technique involves injection of cold saline through a venous line and studying the curve of temperature change detected in the femoral artery. From this curve, the CO is measured, and the ITBV and EVLW can be calculated. These measurements were performed in duplicate to obtain an average score.

At the same time as the TPTD measurements, intra-abdominal pressure was measured and blood samples were obtained to measure lactate levels, central venous saturation of oxygen (SVCO2), and troponin I and natriuretic peptide levels [18]. Intra-abdominal pressure was measured through a catheter with a hydraulic measuring system (Unometer Abdopressure Kit^® ^ConvaTec. Birkerod, Denmark)

The degree of severity of organ dysfunction was assessed by the Sequential Organ Failure Assessment (SOFA score). Tests for other markers (for example, glucose, triglycerides, total and high-density lipoprotein cholesterol, prealbumin, albumin and C-reactive protein levels) were performed. Finally, trans-esophageal echocardiography was performed in patients on mechanical ventilation, and transthoracic echocardiography was performed in non-intubated patients without chest burns. We performed echocardiography on admission (but not after 16 hour) to determine the initial degree of volemia and to exclude other cardiac pathologies. We repeated echocardiography after initial resuscitation (third day ± one day), and within one week (plus or minus one day). To minimize technical variability, only echocardiographic series were included that could be performed during these three stages by cardiologists assigned to the echocardiography unit.

For statistical analyses, we used IBM SPSS Statistics 11.5 software (SPDD Inc., Chicago, IL, USA). Continuous variables are presented as means and standard deviations. Categorical data are summarized as absolute frequencies and percentages. The evolution of parameters over time and the estimated effects of one variable on another were analyzed using mixed models. Correlations between variables were performed by the Pearson or Spearman methods. The Mann-Whitney U-test was used to compare groups of patients. A *P *value < 0.05 was considered to indicate statistical significance.

## Results

Of the 362 burned patients admitted, 132 (36.5%) met the inclusion criteria and had no exclusion criteria. Epidemiological data are shown in Table 1. The mean delay from the burn to monitoring was 4.2 ± 2.8 hours (median, 3.0 hours). After admission, the volume provided to patients (mean 13,058 ± 10,901 mL; 4.75 ± 4.06 mL/Kg/TBSA%) was slightly greater than that predicted by Parkland's formula, but not in the first 8 hours (first 8 hours: 4.05 ± 4.75 mL/kg/TBSA%; 8 to 16 hours: 5.2 ± 4.7 mL/kg/TBSA%, 16 to 24 hours: 5.1 ± 7.0 mL/kg/TBSA%). During the second day, we provided 9,555 ± 4,102 mL (of this a mean of 687 mL were colloids). At 48 hours, the rate of volume provided decreased to 3.0 ± 5.2 mL/kg/TBSA% and at 72 hours, it decreased to 2.8 ± 6.2 mL/kg/TBSA%. The mean hourly urine output was greater than the target (first 8 hours: 0.9 mL/kg/hour; 8 to 16 hours: 1.2 mL/kg/hour; 16 to 24 hours: 1.1 mL/kg/hour; 48 hours: 1.5 mL/kg/hour; and 72 hours: 2.1 mL/kg/hour). Supplementary or total parenteral nutrition was needed by 81 patients.

**Table 1:** Demographic and clinical data of critically burned patients

Variables (number = 132)	Number (%) or Mean ± SD (median)
Male	98 (74.2%)
Female	34 (25.8%)
Another trauma in addition to burns	15 (11.4%)
Mechanism: flame	115 (87.9)
electrical	6 (4.5%)
Location: upper limbs	114 (87.1%)
chest	92 (69.7%)
head and neck	91 (68.9%)
lower limbs	89 (67.4%)
back	44 (33.3%)
buttocks	23 (17.4%)
Inhalation injury: clinical suspicion	52 (39.4%)
subglottic lesions	12 (9.1%)
Age	48 ± 18 (45)
TBSA burned %	35.0 ± 22.1 (28)
ABSI score	8.23 ± 2.66 (8)
Length of ICU stay	27.1 ± 21.8 (22)
Days with mechanical ventilation (number = 99, 75%)	21.5 ± 19.9 (15)
Days with shock (number = 90, 68.2%)	14.8 ± 15.2 (8.5)
Days with ARDS^a ^(number = 32, 24.2%)	7.23 ± 7.61 (4.0)
Days with tracheostomy (*n *= 40, 30.3%)	29.0 ± 21.1 (25)
Days with ARF^b ^(number = 41, 31.1%)	9.66 ± 12.35 (3.0)
Days with CVVHF^c ^(number = 15, 11.4%)	4.46 ± 3.66 (3.0)
SOFA score Day 0	3.69 ± 3.01 (3.0)
Day 1	4.38 ± 3.09 (5.0)
Day 2	4.99 ± 3.05 (6.0)
Day 3	5.14 ± 3.12 (5.0)
Day 5	4.35 ± 3.11 (4.0)
Day 7	3.59 ± 3.07(3.0)
Mortality	31 (23%)

^a^ARDS: as defined the American-European Consensus Conference (AECC) in 1994; ^b^ARF was defined according to the Acute Kidney Injury Network criteria; ^c^CVVHF was initiated according to clinical criteria. Mean ± SD (Median). ABSI, Abbreviated Burn Severity Index; ARDS, acute respiratory distress syndrome; ARF, acute renal failure; CVVHF, continuous venous-venous hemofiltration; SOFA; sepsis-related organ failure; TBSA burned, total body surface area burned;

Hemodynamic measurements showed signs of initial central hypovolemia as follows. The mean ITBVI was <800 mL/m^2 ^and mean arterial blood lactate levels >2.0 mmol/L. Although their initial mean CIs were adequate, 57 patients (43%) had a CI lower than 2.2 L/minute/m^2 ^within the first 24 hours, and in 13 patients the CI remained low after 24 hours. Subsequently, the mean CI significantly increased from initial measurement to 24 hours (*P *= 0.001) and reached a hyperdynamic state. At some point in the resuscitation, 15% of patients required norepinephrine, although most patients were under the influence of sedatives-analgesics, and the norepinephrine doses were less than 0.2 μg/kg/minute. The ITBVI was initially low and increased without reaching supranormal average values. The mean EVLWI remained normal, but had a tendency to rise during the first 48 hours and stabilize on the third day. However, 43 patients (32%) had at least one measurement of EVLWI >10 ml/kg, most frequently at the end of resuscitation. The ratio of EVLW/ITBV may reflect perturbations of pulmonary vascular permeability; however, its mean value was in the normal range (Table 2).

**Table 2:** Hemodynamic, temperature and blood gas measurements

	Initial	24 hours	48 hours	72 hours
HR (bpm)	83 ± 21	95 ± 19	95 ± 19	95 ± 18
Temperature (°C)	35.5 ± 1.8	36.9 ± 1.0	36.6 ± 0.9	36.6 ± 1.3
MAP (mmHg)	85 ± 18	83 ± 13	79 ± 12	78 ± 8
PaO2/FiO2 ratio	338 ± 197	294 ± 114	269 ± 103	292 ± 136
CI (L/min/m^2^)	2.68 ± 1.06	3.22 ± 1.12	3.97 ± 1.12	4.43 ± 0.87
SVI (mL/m^2^)	33.7 ± 13.9	35.1 ± 13.2	42.7 ± 12.6	47.1 ± 10.7
ITBVI (mL/m^2)^	709 ± 254	744 ± 276	823 ± 230	896 ± 214
EVLWI (ml/kg)	6.97 ± 2.56	8.43 ± 4.52	8.85 ± 4.53	8.45 ± 3.80
SVCO_2 _(%)	71.4 ± 8.5	72.5 ± 9.6	75.7 ± 8.7	73.9 ± 8.8
Base deficit	-3.5 ± 4.4	-0.9 ± 3.1	0.9 ± 2.3	2.2 ± 2.7
Arterial blood lactate (mmol/L)	2.58 ± 2.05	2.45 ± 1.78	1.87 ± 1.27	1.46 ± 1.02
Cardiac Troponin-I (ng/mL)	0.14 ± 0.59	0.16 ± 0.45	0.09 ± 0.27	0.07 ± 0.17
NTproBNP (pg/mL)	116 ± 387	280 ± 721	434 ± 961	482 ± 596
IAP (mm Hg)	9.7 ± 4.1	12.1 ± 8.2	11.1 ± 5.3	10.0 ± 3.0
EVLW/ITBV	0.38	0.35	0.31	0.28

bpm, beats/minute; CI, cardiac index; EVLWI, extravascular lung water volume index; HR, heart rate; IAP, intra-abdominal pressure; ITBVI, intrathoracic blood volume index; MAP, mean arterial pressure; NTproBNP, N-terminal pro-brain natriuretic peptide; SVCO_2_, central venous oxygen saturation; SVI, indexed stroke volume.

**Figure 2:**
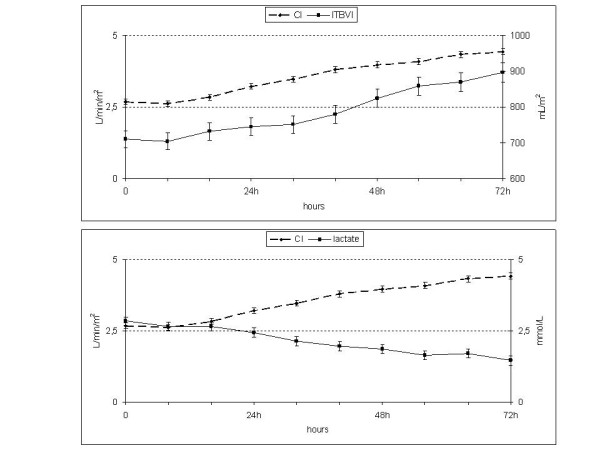
**ITBV, the CI and lactate**. The table shows low initial values of the ITBVI and its progressive elevation, similar to the CI. Lactic acid mirrors the CI (lower panel). With below-normal preload, at 32 hours, the CI and lactate levels were normal. CI, cardiac index; ITBV, intrathoracic blood volume; ITBVI, intrathoracic blood volume index.

In parallel with the increase in the CI and ITBV, there was a progressive decrease in base deficit and arterial blood lactate levels. However, it was not necessary to reach a normal ITBVI to achieve an adequate CI and decrease or normalize lactate levels (Figure 2).

**Figure 3:**
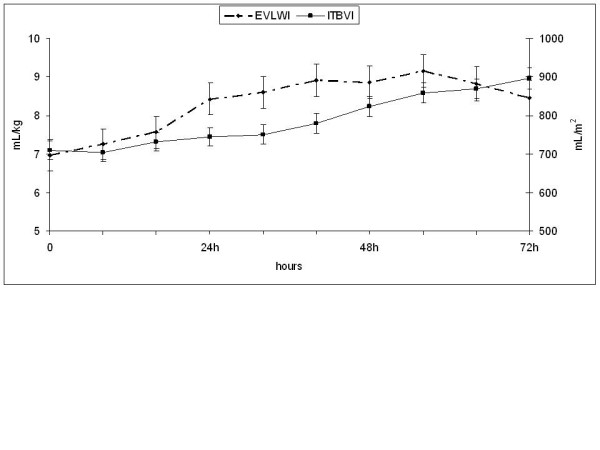
**ITBV and EVLW**. The ITBV index slowly rises while the EVLWI increases, especially during the first 40 hours. EVLWI, extravascular lung water index; ITBV, intrathoracic blood volume.

Therefore, by administering volume for each increment of 100 mL/m^2 ^of ITBVI, a CI increment of 0.17 L/minute/m^2 ^(confidence interval: 0.14, 0.20; *P *= 0.0001) was achieved without an excessive elevation of EVLWI (0.4 mL/kg; 0.2, 0.5; *P *= 0.0001). Similarly, for each L/minute/m^2 ^of CI increment, there was a 0.055 ng/mL decrement in troponin I levels (-0.086, -0.025; *P *= 0.0003), and arterial blood lactate levels decreased by 0.45 mmol/L (-0.56, -0.35; *P *= 0.0001). Finally, an increment of 100 mL/m^2 ^of ITBV had an average positive effect of 56 pg/mL (0.32, 0.80) on the NTproBNP. Therefore, every increase of 100 mL/m^2 ^of ITBVI was associated with a 0.17 L/minute/m^2 ^increase in the CI and a 0.4 mL/kg increase in the EVLWI, while lactate levels decreased by 0.07 mmol/L (Figures 2, 3).

Up to 52 patients had a urine output determination of less than 0.5 mL/kg/hour (42 of these occurred during the first 24 hours) without associated differences in the CI (*P *= 0.4), ITBVI (*P *= 0.2) or lactate levels (*P *= 0.2). In addition, 53 patients (40%) had one or more urine output determinations of >0.5 mL/kg/hour in the presence of hypovolemia as indicated by CI <2.2 L/minute/m^2^, ITBV <800 mL/m^2 ^and lactate levels >2.0 mmol/L. Therefore, there was no relationship between traditional resuscitation indicators (urine output) and those obtained by TPTD. TPTD monitoring could result in changes in resuscitation in 45 patients in the first 48 hours (34%) and in 57 patients (43%) in the first 72 hours.

In patients who underwent echocardiography, the mean CO was within normal limits early in resuscitation and increased thereafter. Left ventricular end-diastolic volume (LVEDV) increased much more during resuscitation than did left ventricular end-systolic volume and was accompanied by a fractional shortening increase. Finally, we found no significant alterations in basic parameters of diastolic dysfunction: ratio of mitral peak velocity of early filling (E) to mitral peak velocity of late filling (A) (E/A), mitral deceleration time and tricuspid annular plane systolic excursion (Table 3). The CI obtained by thermodilution was correlated with that measured by echocardiography (r = 0.59, *P *= 0.007), and although LVEDV and ITBV were initially low, there was little correlation between these two parameters (r = 0.489, *P *= 0.003; rho = 0.313, *P *= 0.07).

**Table 3:** Changes in echocardiographic measurements

	Day 1	Day 3	Day 7
FS (%)	38.6 ± 9.4	41.4 ± 7.4	38.9 ± 6.2
EF. Teich (%)	67.5 ± 12.0	70.2 ± 10.4	67.6 ± 8.48
CO (l/min)	5.18 ± 2.16	6.54 ± 2.44	7.45 ± 2.03
LVESV (ml)	32.0 ± 18.9	32.9 ± 20.3	36.6 ± 17.5
LVEDV (ml)	85.0 ± 36.3	95.6 ± 39.1	110 ± 39.3
E/A	0.98 ± 0.35	1.34 ± 0.57	1.22 ± 0.54
E/E'	6.99 ± 4.37	7.96 ± 4.44	8.77 ± 2.04
Deceleration T (sec)	0.19 ± 0.07	0.18 ± 0.06	0.16 ± 0.05
Isovolumetric relaxation time T (ms)	83.7 ± 32.7	77.9 ± 32.0	74.6 ± 22.9
Lung wave 'a'	23.3 ± 10.0	26.1 ± 9.4	31.1 ± 14.9
TAPSE (mm)	17.5 ± 4.4	20.5 ± 5.8	16.4 ± 6.4

CO, cardiac output; E/A: ratio of mitral peak velocity of early filling (E) to mitral peak velocity of late filling (A); E/E': ratio of mitral peak velocity of early filling (E) to early diastolic mitral annular velocity (E'); EF, ejection fraction; FS, fractional shortening; LVEDV, left ventricular end-diastolic volume; LVESV, left ventricular end-systolic volume; TAPSE, tricuspid annular plane systolic excursion.

**Figure 4:**
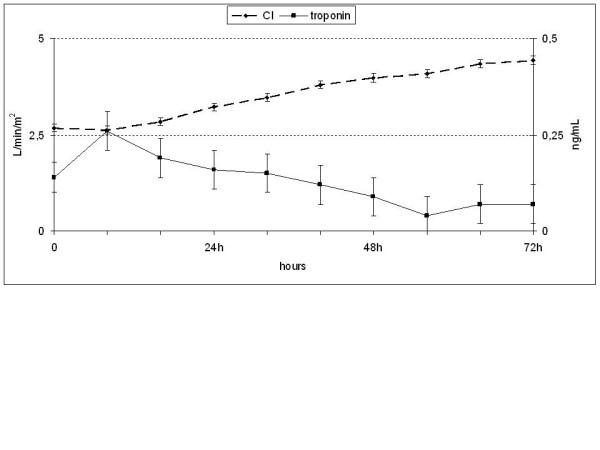
**Troponin I levels**. A small peak of troponin I levels was observed at eight hours with a subsequent decline.

Troponin I was slightly increased, peaking at 8 hours, and then decreased to normal values after 56 hours. SVCO_2_, NTproBNP and heart rate also progressively increased after 16 hours (Figure 4). Although the SVCO_2 _is a global marker of oxygen delivery in real time, it is only an indirect marker of tissue perfusion. In our study, mean saturation values were normal in the early stages, unlike lactate levels, which were high. The SVCO_2 _correlated no better with the CI than did lactate levels (0.452 *P *= 0.001 versus -0480 *P *= 0.000). In addition, like lactate levels, SVCO_2 _did not correlate well with the ITBVI (0.276 *P *= 0.03 versus 0.206 *P *= 0.05) or urine output (0.07 *P *= 0.6 versus 0.07 *P *= 0.07).

There were no differences in the evolution of intra-abdominal pressure, although 16 patients (12%) had at least one determination of intra-abdominal pressure >20 mmHg, and mortality among these patients was 50%.

Finally, we found no relationship between volume requirements within the first 24 hours and mortality, SOFA or days of ICU stay. There was also no clear effect of the specific score (Abbreviated Burn Severity Index) on volume requirements.

## Discussion

Our study evaluated a protocol for resuscitation in a large number of critical burn patients (*n *= 132). In this protocol, fluid therapy was guided by MAP and urine output plus TPTD and tissue perfusion data, but with slightly less restrictive hemodynamic goals than those used by Arlati *et al*. (CI >2.5 instead of 2.2 L/minute/m^2^) [15] and lactate levels were monitored to ensure tissue perfusion. This protocol was shown to be safe and to avoid unnecessary fluid contributions.

We provided less volume than that reported by Holm *et al*. [14] but a similar volume to that calculated by Parkland's formula. However, the infusion rate was lower at the beginning (first eight hours: 4.05 mL/kg/TBSA burned) when patients were in a poor fluid response phase and in which an increase in fluid added only increased the interstitial fluid. We did not find a correlation between the volume administered within the first 24 hours and mortality, organ dysfunction, or length of ICU stay. This finding might be because, in most cases, we avoided an excessive input of fluid.

Hemodynamic data showed that hourly urine output less than 0.5 ml/kg did not necessarily correspond with a low CI, low ITBV or high levels of lactate. Conversely, there was hypovolemia (CI <2.2 L/minute/m^2^, ITBV <800 ml/m^2 ^and lactate levels >2.0 mmol/L) in many patients with an hourly urine output >0.5 ml/kg. Therefore, our data corroborate previous studies showing that urine output and vital signs, such as blood pressure and heart rate, are insufficient to guide resuscitation of critically burned patients [13,20,21].

In our study, we observed initial central hypovolemia, manifested by a low CI, ITBV, and LVEDV and high lactic acid, with a poor response to initial fluid resuscitation. After the first 24 to 48 hours, the CI and preload normalized and no new episodes of hypovolemia were observed. These results are similar to those reported by other authors who provided even larger quantities of volume, yet also failed to correct the signs of central hypovolemia within the first 24 hours [14,22]. In recent years, resuscitation guided by hemodynamic parameters and the increased use of opioids has led to the administration of more volume, a phenomenon that has been termed fluid creep [23-26]. Although some authors have reported that increased fluid intake can decrease mortality [27], others have failed in their attempts to correct the initial hypovolemia and morbidity and mortality with increased fluid intake [14,15]. Conversely, it is believed that increased edema may intensify burns, facilitate infection and increase the risk of multiple organ dysfunction [2,3].

The ITBVI was correlated with the CI and, usually, an increase in the CI corresponded with an increase in the ITBVI. Some studies attempted to achieve targets of an ITBVI greater than 800 mL/m^2 ^and CI >3.5 L/minute/m^2^, leading to large volumes being provided to patients without finding significant differences in preload or CO parameters with those in whom resuscitation was performed guided by Parkland's formula [14,28]. For this reason, we adopted objectives similar to those used by Arlati *et al*. [15], and we accepted CI values of at least 2.5 L/minute/m^2^. We then observed that with values of an ITBVI less than 800 ml/m^2^, it was possible to reach an adequate CI and decreased lactate levels. Therefore, we consider that the goal of resuscitation should be to get the appropriate CI and lactate levels. Attempting to normalize preload during an increase in the permeability phase does not always produce persistent hemodynamic improvement, and yet it increases the interstitial fluid.

Another factor useful in the management of fluid is EVLW [29,30]. Holm *et al*. [31] found a progressive increase in EVLW in burn patients during the resuscitation and Arlati *et al*. [15] in the Parkland resuscitation group found the same. Instead, this was not observed in the permissive hypovolemia group in Arlati *et al*.'s study [15] or in a study by Bak [10]. We found normal initial levels of EVLW and a progressive increase during the first 40 hours of resuscitation. High EVLW/ITBV may reflect a disturbance of vascular permeability. In our study, EVLW/ITBV was high during the first resuscitation day, although this may not indicate an alteration of permeability and could be mainly attributed to hypovolemia, rather than an increase in EVLWI. However, on the second day, the permeability could have been altered because the EVLWI was increased, even with a low ITBVI. During the third day, the permeability appeared to decrease. However, most of the EVLWI values in excess of 10 mL/kg were observed on the third day. Other factors that may have influenced these findings include lymphatic drainage, which can prevent an initial increase in the EVLWI and allow its elevation when saturated [31,32]. Therefore, EVLW increases to provide volume during resuscitation and may provide useful data for limiting volume input during this stage.

In our study, the echocardiographic data were similar to those obtained by other groups with regard to low CO and low initial blood volume (low LVEDV) and the subsequent elevation of both parameters. In contrast, we did not find any changes in cardiac relaxation patterns in the initial phase, with a mitral deceleration time of 0.19 ± 0.07 seconds [33,34], or a good correlation between LVEDV and ITBV [10,35]. Therefore, considering that echocardiography requires expert personnel who are not always available, the transesophageal technique has limitations, chest burns hinder transthoracic echocardiography, and echocardiography is more complicated than TPTD, we propose that echocardiography should be performed at admission with subsequent monitoring performed by TPTD.

In critically burned patients, moderate elevations of troponin I have been observed, and a certain degree of injury has been suggested, even with hyperdynamic cardiac function during resuscitation [17]. We found that troponin I levels were 0.14 ng/mL at admission, peaked at eight hours (0.26 ng/mL) and progressively declined thereafter. Because troponin I increases three hours after myocardial damage and peaks at twelve hours, we conclude that the limited changes that occurred in our patients might have been due to an initial myocardial insult, and that the amount of fluid provided during periods of hypovolemia had no effect.

The limitations of this study are that it was conducted at a single center and that the percentage of change in fluid rate was performed at the discretion of clinicians without specific goals. However, the results appear to indicate that the objectives were met. Another limitation is that lactate levels may not reflect changes in real time. Factors, such as mechanical ventilation, sedatives-analgesics or surgery, may influence hemodynamic changes, but are inevitable confounders in this clinical setting. Data on length of stay and overall mortality are not particularly valuable, because they are affected by other conditions, such as an outbreak of multiresistant Klebsiella [36].

Hemodynamic changes consisting of low CO and hypovolemia with poor responsivity to fluid occur during the first 12 to 36 hours. MAP and diuresis insufficiently reflect central blood volume. Therefore, additional monitoring with TPTD and biochemical parameters is necessary. Given the absence of severe myocardial injury, and that increasing flow rate during the initial resuscitation phase can lead to interstitial fluid accumulation with little impact on blood volume, we propose that the objective should not be to normalize parameters, but rather to ensure a minimum CI in the absence of poor perfusion data. The ITBVI should be used as an indicator of the volume required and the efficiency of its administration. Elevation in EVLW and the ITBVI can be used as limits for the prevention of pulmonary edema.

## Conclusions

Central hypovolemia occurs during the resuscitation phase and may not be reflected by MAP or urine output, although it is reliably detected by TPTD. The initial hypovolemia cannot always be corrected immediately, but it is possible to achieve an adequate CI and tissue perfusion with below-normal levels of preload. Early resuscitation guided by lactate levels and hemodynamic targets below normal appears safe and avoids unnecessary fluid input.

## Key messages

• Hemodynamic changes in severely burned patients consist of low CO and hypovolemia with poor responsivity to fluid during the first 12 to 36 hours.

• It is possible to reach an adequate CI and decreased lactate levels with ITBVI values less than 800 ml/m^2^.

• Urine output and vital signs are not suitable for monitoring resuscitation in severe burns.

• Early resuscitation guided by lactate levels and hemodynamic targets below normal appears safe and avoids unnecessary fluid input

## Abbreviations

CI: cardiac index; CO: cardiac output; E/A: ratio of mitral peak velocity of early filling (E) to mitral peak velocity of late filling (A); E/E': ratio of mitral peak velocity of early filling (E) to early diastolic mitral annular velocity (E'); EVLW: extravascular lung water; EVLWI: extravascular lung water index; IAP: intraabdominal pressure; ITBV: intrathoracic blood volume; ITBVI: intrathoracic blood volume index; LVEDV: left ventricular end-diastolic volume; MAP: mean arterial pressure; NTproBNP: N-terminal pro-brain natriuretic peptide; SOFA: sequential organ failure assessment; SVCO_2_: central venous saturation of oxygen; TBSA: total body surface area; TPTD: transpulmonary thermodilution.

## Competing interests

The authors declare that they have no competing interests.

## Authors' contributions

MS conceived the study and participated in its design, collected the data and drafted the manuscript. AGL conceived the study, participated in its design and coordination, as well as prepared the manuscript. EH collected data and participated in manuscript preparation. TL carried out echocardiography studies and helped to draft the manuscript. BG collected data and participated in manuscript preparation. MJA collected data and participated in manuscript preparation. LC collected data and participated in manuscript preparation. CC participated in the design of the study and prepared the manuscript. All authors read and approved the final manuscript.
